# Key Immune Events of the Pathomechanisms of Early Cardioembolic Stroke: Multi-Database Mining and Systems Biology Approach

**DOI:** 10.3390/ijms17030305

**Published:** 2016-02-27

**Authors:** Chia-Chou Wu, Bor-Sen Chen

**Affiliations:** Laboratory of Control and Systems Biology, Department of Electrical Engineering, National Tsing Hua University, Hsinchu 30013, Taiwan; d9761820@oz.nthu.edu.tw

**Keywords:** systems biology, cardioembolic stroke, inflammation, immune system, functional network, core network

## Abstract

While inflammation has generally been regarded as a negative factor in stroke recovery, this viewpoint has recently been challenged by demonstrating that inflammation is a necessary and sufficient factor for regeneration in the zebrafish brain injury model. This close relationship with inflammation suggests that a re-examination of the immune system’s role in strokes is necessary. We used a systems biology approach to investigate the role of immune-related functions via their interactions with other molecular functions in early cardioembolic stroke. Based on protein interaction models and on microarray data from the blood of stroke subjects and healthy controls, networks were constructed to delineate molecular interactions at four early stages (pre-stroke, 3 h, 5 h and 24 h after stroke onset) of cardioembolic stroke. A comparative analysis of functional networks identified interactions of immune-related functions with other molecular functions, including growth factors, neuro/hormone and housekeeping functions. These provide a potential pathomechanism for early stroke pathophysiology. In addition, several potential targets of miRNA and methylation regulations were derived based on basal level changes observed in the core networks and literature. The results provide a more comprehensive understanding of stroke progression mechanisms from an immune perspective and shed light on acute stroke treatments.

## 1. Introduction

Proinflammatory cytokine in mice has been shown to be a negative regulator of progenitor proliferation [1], which is a critical step in brain regeneration in the zebrafish model [2]. Nevertheless, inflammation was shown to be necessary and sufficient for enhancing the proliferation of neural progenitors and subsequent neurogenesis [3]. Since proinflammatory cytokines can promote inflammation, these findings have spurred debates about the role of inflammation in stroke recovery, and more comprehensive studies into the relationship between inflammation and stroke recovery are thus required. The close relationship between inflammation and the immune system indicates that the role of the latter in strokes is worth re-examining from a systems biology perspective. A recent genome-wide high-throughput experiment examined patients with cardioembolic (CE) stroke at ≤3 h, 5 h and 24 h after stroke onset and compared this group with a vascular risk factor control group of patients without symptomatic vascular diseases [4]. Although this study uncovered some significant differences in the expression of genes related to cell death, coagulation and inflammatory pathways, the roles of inflammation and immune responses in CE stroke remain to be elucidated. The present study therefore carried out a further exploration of the pathophysiology of ischemic stroke by investigating the roles of immune-related molecular mechanisms and their relationships with other molecular functions in early CE stroke.

Systemic inflammation is linked to the occurrence of strokes and may involve not only peripheral cells, such as leukocytes, but also brain cells, such as glia, endothelial cells and neurons [5]. Recent evidence suggests that elements of the immune system are intimately involved at all stages of an ischemic cascade, from the acute intra-vascular events triggered by the interruption of the blood supply to the parenchymal processes leading to brain damage and the ensuing tissue repair [6]. The interactions between innate immune cells in the brain have also become better understood in recent years, prompting the realization that each of these cell types contributes to the development of inflammation in the brain [7]. Multi-protein complexes, known as inflammasomes, process damage-associated molecular patterns to trigger an effector response [8]. The pathophysiological processes following strokes are complex and extensive and include bioenergetic failure, loss of cell ion homeostasis, acidosis, increased intracellular calcium levels, excitotoxicity, reactive oxygen species-mediated toxicity, generation of arachidonic acid products, cytokine-mediated cytotoxicity, activation of neuronal and glial cells, complement activation, disruption of the blood-brain barrier and infiltration of leukocytes [8]. Intravenous recombinant tissue plasminogen activator (r-tPA), used to induce thrombolysis following a thrombotic occlusion, is currently the only pharmacological agent approved for acute stroke therapy [5]. However, a major limitation of tPA therapy is its narrow therapeutic window of 3–4.5 h [6,8]. A better understanding of the processes involved is therefore urgently required to develop enhanced therapies. One approach is the construction of the underlying molecular interaction networks based on database mining and high-throughput datasets from the blood of normal and stroke subjects, which can be used to study the differences between pre- and post-stroke, pre- and post-treatment and the effects of the standard tPA treatment.

The present study first focused on those immune-related functions (e.g., B- and T-cell activation, inflammation, interleukin signaling pathway, *etc*.) that were significantly enriched in constructed protein–protein interaction networks (PPINs). The interactions of inflammation- and immune-related functions with other significantly enriched functions (e.g., ubiquitin proteasome pathway, multiple growth factors pathways, *etc*.) in the constructed networks are then identified at different stages. Using network comparisons between the functional networks, the interactions of immune-related functions with other functions in the early stages of CE stroke are discussed, and their roles in the mechanisms of stroke progression are explored. In addition, proteins with estimated basal level changes in the core networks at different stages are used to investigate the roles of miRNA and methylation regulations in stroke pathogenesis. Finally, several potential druggable targets are proposed based on their importance in the core networks and on the literature. The results provide a more comprehensive understanding of stroke pathophysiology from the perspective of systems biology and shed light on the development of targeted therapy for strokes based on core network markers.

## 2. Results and Discussion

### 2.1. Network Summary

This study aimed to explore the pathomechanisms of ischemic stroke by investigating the roles of immune-related functions and their relationships with other molecular functions after cardioembolic (CE) stroke. To this end, we first utilized microarray data and protein interaction models to construct protein–protein interaction networks (PPINs) and then compared them to examine changes in functional and core networks during early CE stroke pathogenesis. In network construction, microarray data (GSE58294, [4]) was used to identify the interaction activities between proteins (see Material and Methods section for details). Four different PPINs were constructed based on microarray data for four corresponding stages of CE stroke (C: control; I: ≤3 hps; II: 5 hps; III: 24 hps). The basic information for the constructed networks is shown in Table 1. It should be noted that all genes and their expression profiles were used to obtain the resultant networks, in contrast to a previous procedure [9,10] in which differentially expressed genes (DEGs) are selected. PPINs in the present study thus represent networks of all proteins, while the previously published PPINs are networks of proteins with significantly differential levels of expression only. Since there are proteins that do not typically show significant changes in intracellular levels but do play an important role under altered conditions, we suggest that our more comprehensive approach to network construction is more appropriate. In each constructed network, about 9% of nodes have significantly differential expression (Bonferroni-corrected *p*-value ≤0.05) during early CE stroke, which implies that about 90% of nodes are neglected when only DEGs are considered.

Using a bioinformatics classification system and principal network projection, each of the constructed networks can be presented at two different granular levels: a network of enriched functions and a network of core proteins. In total, 38 enriched functions are included in the four constructed networks (see Table S1 in supplement file for the function names and members in each function). Four groups are used to categorize these enriched functions based on their biological significance: immune, neuro/hormone, growth/death, and general pathways. Inflammation, interleukin, B and T cell activation, and toll-like receptors (TLRs) belong to the immune group; neurodegenerative diseases, dopamine, corticotropin, endothelin, and acetylcholine-related pathways belong to the neuro/hormone group; multiple growth factor-related pathways and apoptosis belong to growth/death group, and ubiquitin, G-protein coupled receptor, transcription, and integrin-related pathways belong to the general pathways group. The presence of functions and the changes in interaction between them in a comparison of the functional networks at different stages can provide guidance as to the roles played by the enriched functions in the pathomechanisms of early CE strokes.

In addition to functional networks, principal network projection is used to extract the main features of the constructed networks. Inspired by image compression and facial recognition, singular value decomposition (SVD) is used to extract so-called “eigen-interactions” which can be used to represent the majority of the interactions in the constructed networks. Core proteins whose interactions have high similarity to the principal eigen-interactions are then used to form core networks at each stroke stage. A comparative analysis of stage-specific core networks allows the identification of key molecules in the progression of CE strokes and their evaluation as potential drug targets. The goal of explaining the functional and molecular mechanisms in the early pathogenesis of CE strokes can thus be achieved using this approach.

### 2.2. Changes in Functions and Proteins Immediately after CE Strokes

**Figure 1:**
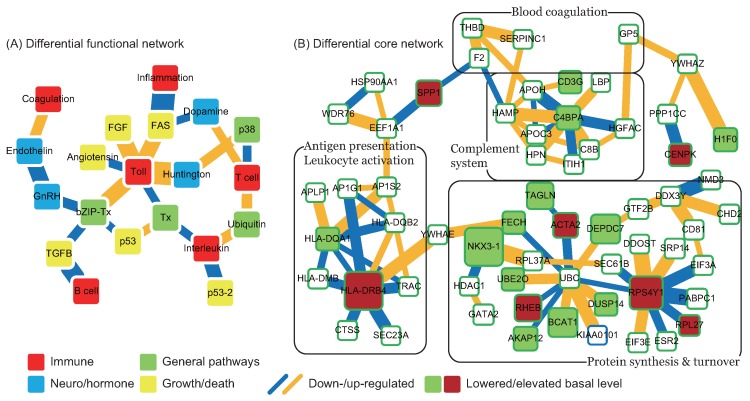
Differential functional and core networks from Stage C to I. (**A**) Differential functional network. Node colors indicate the biological significance of the enriched functions. Red: immune; blue: neuro/hormone; green: general pathway; yellow: growth/death. Colors of links indicate changes in interaction ability from Stage C to I. Blue: down-regulated; orange: up-regulated. Link width indicates the absolute value of the difference of interaction ability from Stage C to I; (**B**) Differential core network. Node colors indicate changes in basal level, representing changes in miRNA and methylation regulations from Stage C to I. Green: lowered; red: elevated. Link colors indicate changes in interaction ability from Stage C to I. Blue: down-regulated; orange: up-regulated. Link width indicates the absolute value of the difference in interaction ability from Stage C to I.

By comparing the functional network of Stage I with Stage C, we obtained the differential functional network for C to I (Figure 1A). Blood coagulation and the endothelin signaling pathway are conspicuous for their roles in vascular regulation. Although the role of the endothelin signaling pathway in the pathogenesis of CE stroke is unclear, endothelin 1 is involved in the regulation of basilar constriction, and dysregulation of basilar artery function may worsen stroke injury [11]. While blood coagulation is detrimental to stroke patients [12], a coagulation cascade can activate inflammatory and immune responses. The interleukin (IL) signaling pathway (IL1, IL2, IL6, and IL10 are found in the constructed networks) is associated with the TLR signaling pathway and T cell activation via general transcription regulation and the ubiquitin proteasome pathway, respectively, which explains the role of interleukins in the activation of inflammation and the immune-related response following stroke onset. The TLR signaling pathway has direct links to Huntington’s disease; so does T cell activation, via the p38 MAPK pathway. This indicates that the interleukin signaling pathway plays a role in neuroprotective processes post-stroke [13]. The TLR signaling pathway interacts with angiotensin II-stimulated signaling and the fibroblast growth factor (FGF) signaling pathway. Angiotensin II is a major causative factor in the cerebrovascular effects of hypertension [14], which has a down-regulated interaction with the TLR signaling pathway. The FGF signaling pathway signifies a good prognosis [15] and may lead to angiogenesis and neuro-protection after strokes [16]. Both the TLR signaling pathway and T cell activation interact with the FAS signaling pathway, which has negative effects on neuroprotection and causes cell death. The tight regulation of the FAS signaling pathway by inflammation- and immune-related pathways is apparent in this differential functional network. The transforming growth factor (TGF)-*β* signaling pathway has a down-regulated interaction with B cell activation, which indicates that the ability of TGF-*β* signaling to limit inflammation is reduced after strokes. In summary, after CE stroke the inflammation- and immune-related pathways are interwoven with neurodegeneration and cell death pathways and exert a combination of adverse and beneficial actions.

In addition to the differential functional network for C to I, the differential core network can further reveal the molecular mechanisms that operated immediately after strokes. In contrast to the functional networks, node color in the diagrammatic representation of differential core networks (Figure 1B) indicates changes in the basal level ($\beta_{i}$) of proteins (green and red indicate lowered and elevated basal levels, respectively). The proteins F2, GP5, SERPINC1, and THBD, which are connected to blood coagulation, bridge the complement system to other proteins, including SPP1 and YWHAZ. SPP1 is a cytokine and can activate interferon *γ* (IFN*γ*) and IL12. SPP1 also links to a group of proteins related to the antigen-presenting process and T cell activation, *i.e.*, the HLA protein family. YWHAZ and YWHAE, two general signal transduction proteins belonging to the 14-3-3 protein family, are involved in the FGF signaling pathway and Parkinson’s disease. In particular, YWHAE bridges the antigen-presenting process and the control of protein synthesis and turnover. In the group of proteins controlling protein synthesis, function, and turnover, RPS4Y1, a cytoplasmic ribosome, is a protein product of a Y-linked gene. It and its interchangeable counterpart, RPS4X, are over-expressed in new-onset heart failure [17], which helps explain the existence of RPS4Y1 at the onset of CE strokes and in particular its higher basal level post-stroke. Not surprisingly, several proteins involved in the regulation of transcription and translation are present (EIF3E, EIF3A and GTF2B), as are several ribosome proteins related to protein synthesis. The ubiquitin proteasome pathway not only controls protein synthesis and turnover but also participates in neurodegenerative diseases [18]. UBC (Ubiquitin C), the central protein in the ubiquitin proteasome pathway, interacts with several proteins related to inflammation (ACTA2), the heme synthesis pathway (FECH), the TGF-*β* signaling pathway (DUSP14), and the PI3K-Akt signaling pathway (RHEB). The ubiquitin proteasome pathway is thus involved in protein synthesis and the turnover of several functions that are critical to stroke status immediately after stroke onset. In summary, the differential core network revealed large changes immediately after stroke onset in the interactions between and basal levels of inflammation- and immune-related functions, as well as in UBC- and RPS4Y1-related protein synthesis and turnover.

### 2.3. Changes in Functions and Proteins after Tissue Plasminogen Activator Treatment

**Figure 2:**
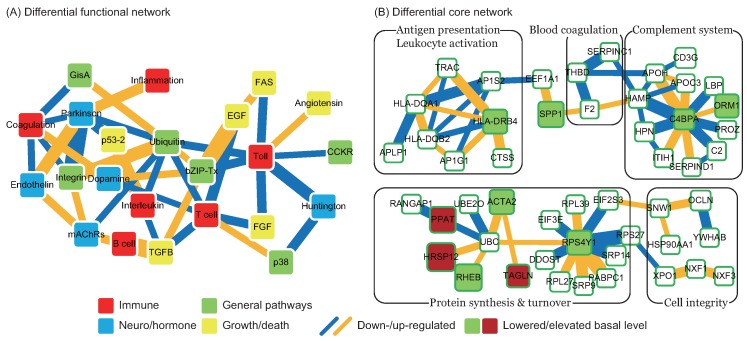
Differential functional and core networks from Stage I to II. (**A**) Differential functional network. Node colors indicate the biological significance of the enriched functions. Red: immune; blue: neuro/hormone; green: general pathway; yellow: growth/death. Link colors indicate changes in interaction ability from Stage I to II. Blue: down-regulated; orange: up-regulated. Link width indicates the absolute value of the difference in interaction ability from Stage I to II; (**B**) Differential core network. Node colors indicate changes in basal level, representing changes in miRNA and methylation regulations from Stage I to II. Green: lowered; red: elevated. Link colors indicate changes in interaction ability from Stage I to II. Blue: down-regulated; orange: up-regulated. Link width indicates the absolute value of the difference in interaction ability from Stage I to II.

By comparing the functional network of Stage II with Stage I, we obtained a differential functional network for I to II (Figure 2A). The major difference between the two stages is the application of tPA treatment: Stage I is untreated and Stage II is treated. The immediate effects of tPA treatment on the functions and proteins can be observed in the differential functional and core networks for I to II. In the differential functional network for I to II, almost all interactions between functions display reverse changes. This includes blood coagulation and the endothelin signaling pathway, the interleukin signaling and ubiquitin proteasome pathway, T cell activation and the ubiquitin proteasome pathway, Huntington disease and the TLR pathway, the TLR and FAS signaling pathway, *etc*. The tPA treatment not only alters the direction of interaction changes but also strongly enhances the effects of inflammation- and immune-related functions, *i.e*., more functions are connected to these functions and more interactions are added between the enriched functions. Moreover, blood coagulation following tPA treatment has connections to the dopamine receptor-mediated signaling pathway and Parkinson’s disease, both of which are related to neurodegenerative diseases. The treatment can cause hemorrhagic side effects [19], which can be explained by the up-regulated interaction between the TLR and angiotensin signaling pathways, causing blood vessel instability. The integrin signaling pathway, which plays a role in vascular stability, interacts with Parkinson’s disease, blood coagulation, and the ubiquitin proteasome pathway. The interactions of the ubiquitin proteasome pathway with the TLR signaling pathway, T cell activation, and the interleukin signaling pathway are down-regulated. This may be detrimental to the regeneration-promoting ability of inflammation- and immune-related functions. In summary, tPA treatment was shown to reverse most of the trends in interaction activity changes post-stroke, but may also cause a worsened prognosis.

The differential core network for I to II is shown in Figure 2B, with node and edge styles as in Figure 1B. The main difference between these two core networks is the separation of HLA-DRB4 from UBC- and RPS4Y1-related functions, caused by the absence of YWHAE and FECH. YWHAE belongs to the FGF signaling pathway and Parkinson’s disease, and FECH is related to iron regulation. While UBC- and RPS4Y1-related functions continue interacting with a similar set of proteins as in the C to I network, most of the interacting proteins show changes in basal level ($\beta_{i}$), e.g., RHEB, ACTA1, TAGLN, and RPS4Y1. Basal level changes of these four proteins cause a dramatic change in protein synthesis and turnover following tPA treatment. In summary, tPA treatment was found to affect the integrity of the core network and to reverse basal level changes in comparison to the differential core network for C to I.

### 2.4. Changes in Functions and Proteins in Early tPA Treatment

**Figure 3:**
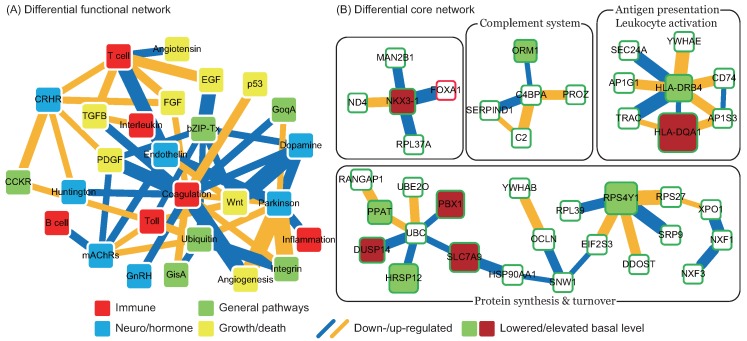
Differential functional and core networks for Stage II to III. (**A**) Differential functional network. Node colors indicate the biological significance of the enriched functions. Red: immune; blue: neuro/hormone; green: general pathway; yellow: growth/death. Link colors indicate changes in interaction ability from Stage II to III. Blue: down-regulated; orange: up-regulated. Link width indicates the absolute value of the difference in interaction ability from Stage II to III; (**B**) Differential core network. Node colors indicate changes in basal level, representing changes in miRNA and methylation regulations from Stage II to III. Green: lowered; red: elevated. Link colors indicate changes in interaction ability from Stage II to III. Blue: down-regulated; orange: up-regulated. Link width indicates the absolute value of the difference in interaction ability from Stage II to III.

By comparing the functional network of Stage III with Stage II, we obtained a differential functional network for II to III (Figure 3A). Since the major difference between the functional networks for these stages is time after tPA treatment, the differential network can be used to assess the effects of early tPA treatment. A reverse trend in interaction changes between enriched functions in comparison to the differential core network for I to II demonstrates a decay in treatment effect over time. The emergence of the Wnt signaling pathway and the platelet-derived growth factor (PDGF) signaling pathway is noteworthy, because of their roles in neuroprotection, regeneration, and vascular growth. In this network, interactions of the Wnt signaling pathway with the TLR and endothelin signaling pathways are up-regulated, while interactions with the dopamine receptor-mediated signaling pathway and transcription regulation by bZIP transcription factor are down-regulated. These findings support the interpretation that the Wnt signaling pathway plays a role in immune-related functions and neurodegenerative diseases [20]. The PDGF signaling pathway has down-regulated interactions with transcription regulation by bZIP transcription factor, blood coagulation, and the muscarinic acetylcholine receptor (mAChR) signaling pathway, and up-regulated interactions with the corticotropin-releasing factor receptor (CRFR) signaling pathway. This indicates that the PDGF signaling pathway may be a critical mechanism for the effectiveness of tPA treatment [21] and that the mAChR and CRFR signaling pathways may employ different mechanisms to achieve their neuroprotective roles. In summary, tPA treatment was shown to combine several functions to achieve its therapeutic effect over time, and to establish tight connections between these functions.

**Figure 4:**
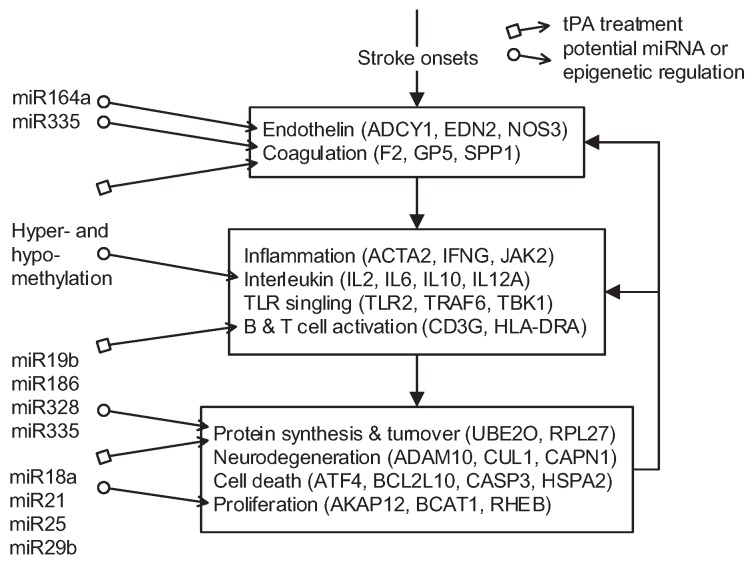
Diagram of the pathomechanism of early CE stroke and the enriched functions regulated by tPA treatment and miRNAs, showing functions and proteins affected after CE stroke onset. Symbol arrows indicate where tPA treatment (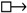) and miRNA regulation (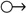) can interfere with the progression of stroke and potential targeted functions and proteins.

As described above, the differential core network for II to III (Figure 4B) reveals the molecular mechanisms of early tPA treatment, illustrating how the tPA takes effect after treatment for 20 h. The network retains the same components (HLA-DRB4, C4BPA, UBC, and RPS4Y1) as the previous differential core networks, but these proteins are now disconnected. Several key molecules can be discerned in this network. Antigen presenting-related HLA class II proteins HLA-DRB4 and HLA-DQA1 have been implicated in heart disease and ischemic stroke [22,23]; the specifics of how tPA treatment affects HLA class II proteins are still unclear, however. ORM1, an acute phase plasma protein, is present at increased levels due to acute inflammation; its basal level and down-regulated interaction with C4BPA, a multimeric protein that controls activation of the complement cascade, may be a result of the decayed effectiveness of tPA [24]. FOXA1 and NKX3-1 are two transcription factors active in prostate tumor progression through collaboration with androgen receptor (AR), which is neuroprotective in strokes [25]. Instead of a direct interaction, UBC and RPS4Y1 are connected through EIF2S3, SNW1, HSP90AA1, and SLC7A9. SNW1 is involved in the notch signaling pathway, which can cause an unusual susceptibility to strokes [26] and promotes cell proliferation and differentiation after strokes [27]. SLC7A9 mediates the transport of cysteine and can control the level of homocysteine in the blood, which is an indicator for vascular diseases and stroke [28]. In summary, the differential core network for II to III was shown to become more broken up than the previous ones and indicates the more extensive range affected by strokes and tPA treatment.

### 2.5. Pathomechanisms and Potential Drug Targets

The comparative analysis of the functional and core networks provides insights into the pathomechanisms of EC stroke and how standard tPA treatment affects stroke progression. Figure 4 summarizes the findings of this study. After the onset of CE stroke, changes in blood vessels activate the endothelin and blood coagulation functions. Via interactions with the coagulation cascade, inflammation- and immune-related functions are activated to rectify the abnormal status caused by an obstruction in a blood vessel. These function as compensators attempting to rectify the abnormality by interacting with protein synthesis and turnover, neurodegeneration, cell death, and proliferation. However, these interactions are not always beneficial for post-stroke recovery; some neurodegenerative diseases in particular are connected to inflammation- and immune-related functions [29]. Moreover, endothelin, blood coagulation, and inflammation- and immune-related functions are subject to feedback from protein synthesis and turnover, cell death, and proliferation. Under tPA treatment, blood coagulation, B and T cell activation, and protein synthesis are affected (indicated by 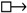 in Figure 4), and the pathomechanism of CE stroke is also subject to interference. Although this interference can briefly relieve the symptoms caused by vessel obstruction, several neurodegenerative diseases emerge in the functional networks following tPA treatment.

In addition to the pathomechanisms that govern how CE strokes and tPA treatment affect the physiology, a comparison of protein basal levels between subsequent stages also provides an insight into microRNA (miRNA) and methylation regulation in stroke pathophysiology. Recent studies indicate that alterations in miRNA expression respond to ischemic stroke in animal models [30]. The miRNA–target pairs [31] consistent with a dysregulation of miRNA following ischemic stroke [32] resulting from our comparative core network analysis are summarized in Table 2. These potential miRNA/epigenetic regulations of the enriched functions are also indicated in Figure 4 (indicated by 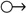). In addition to miRNA regulation of protein basal levels, methylation regulation is a potential mechanism that can change protein basal levels after stroke onset. Although studies of methylation in strokes have indicated a range of changes in protein basal level [33], the targets of methylation regulation have not yet been the subject of a dedicated study. Proteins that have large basal level changes and are not miRNA targets (ACTA2 [34], C4BPA [35], CD3G [36], CENPK [37], DEPDC7 [38], FECH [39], HLA-DQA1 [40], HLA-DRB4 [41], and NKX3-1 [42]) can be potential targets of methylation regulations. Based on the specific targets of miRNA regulations and the position of the target proteins in the core networks, several potential drug targets can be selected (* in Table 2). SPP1 in blood coagulation bridges the complement systems and antigen presentation, and the connection can activate the subsequent inflammation and immune responses. In addition, SPP1 can also activate IFN*γ* and IL12, making it a potential treatment candidate. Another potential target is RPS4Y1, a male-specific protein that may play a role in males’ higher susceptibility to stroke. Finally, the possibility that the targets of miRNA and methylation regulation are identical cannot be ruled out and there are other factors may cause the changes of protein basal levels, such as differential gene regulations through transcription factors. The mechanisms of miRNA and methylation regulations after CE stroke onset require further investigation in future studies.

## 3. Material and Methods

**Figure 5:**
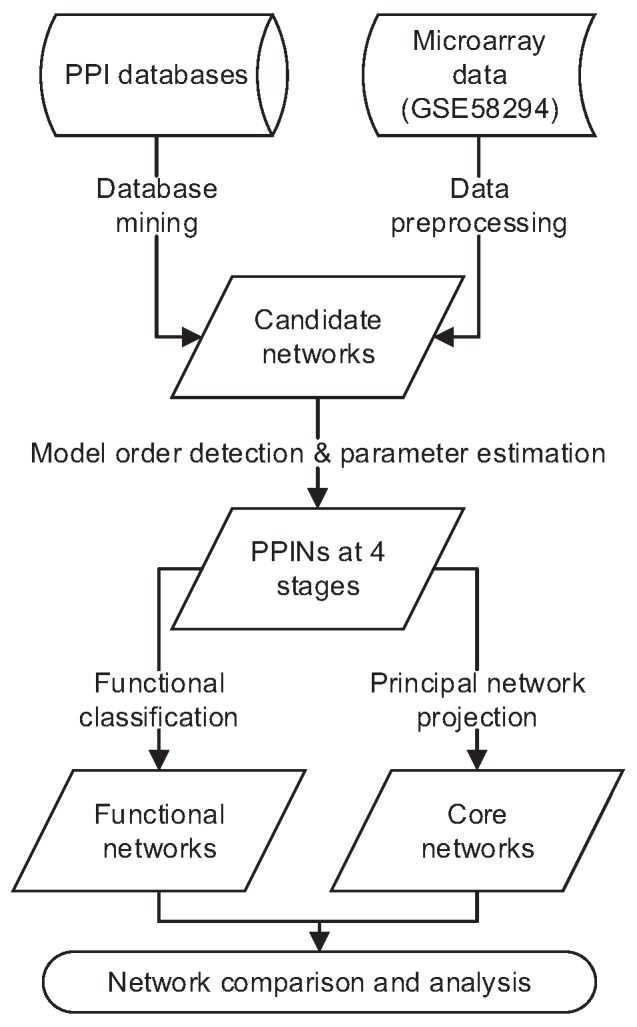
Flowchart of the early cardioembolic (CE) stroke model analysis process, consisting of data preprocessing, interaction network construction, principal network projection and comparative analysis of functional and core networks.

The analysis workflow (microarray data preprocessing, interaction network construction, principal network projection and comparative network analysis) is summarized in Figure 5.

### 3.1. Microarray Data for Early Cardioembolic Stroke

The microarray dataset for early cardioembolic stroke (Gene Expression Omnibus (GEO) Accession No. GSE58294 [4]) contains gene expression data from the blood of subjects with CE stroke and of a vascular risk factor control group without symptomatic vascular diseases. We assayed 23 control samples (C) and 23 cardioembolic stroke samples for each of three time points (*i.e*., ≤3 (I), 5 (II) and 24 (III) hours post-stroke (hps)). GC robust multi-array average-empirical-Bayes (GCRMA-EB) background adjustment, quantile normalization and median-polish summarization were performed on the raw data (CEL files) using MATLAB^®^ (The MathWorks Inc., Natick, MA, USA).

### 3.2. Network Construction

The microarray data processing yielded 23,520 gene expression levels at four stages (C, I, II, III). Owing to the computational complexity of considering all interactions among all proteins, candidate interactions mined from protein-protein interaction (PPI) databases were used as candidates for the subsequent network construction. Since the candidate network considered for the CE stroke condition contained many false positive interactions, further pruning using microarray data was necessary to complete network construction. The details are described in the following sections.

#### 3.2.1. Candidate Network Construction via Multi-Database Mining

To reduce computational complexity, candidate PPIs had to be provided prior to identifying interaction activities in the protein interaction model. These PPI candidates were collected from 10 frequently-used PPI databases (BIND [43], BioGRID [44], DIP [45], HPRD [46], I2D [47], IntAct [48], MINT [49], PIP [50], Reactome [51] and STRING [52]) and consisted of interactions based on computational predictions and biological experiments. Candidate PPIs were then pruned using the corresponding microarray data at different stages of CE stroke to construct realistic stage-specific PPI networks. The intersection of the collected candidates and the genes recorded in microarray data yielded a candidate network containing 15,017 proteins and 319,362 interactions.

#### 3.2.2. Protein Interaction Model

We then introduced a protein interaction model to describe the PPIs at a specific stage (labeled C, I, II or III). Assuming there are *P* proteins in the candidate network (p= 15,017 in this study), the interactions of a target protein *i* with other proteins in the *m*-th sample can be formulated as follows:

(1) $$y_{i}\left(m\right)=\sum_{k=1}^{P}\alpha_{ik}y_{k}\left(m\right)+\beta_{i}+\epsilon_{i}\left(m\right)$$

where $y_{i}\left(m\right)$ is the level of target protein *i* in the *m*-th sample; $\alpha_{ik}$ is the interaction activity of target protein *i* with interacting protein *k*; $y_{k}\left(m\right)$ is the level of protein *k* in the *m*-th sample ($\alpha_{ik}=0$ if there is no interaction between protein *k* and target protein *i*, or i=k, *i.e*., that protein has no self-regulation); $\beta_{i}$ is the basal level of target protein *i* ($\beta_{i}\geq 0$); and $\epsilon_{i}\left(m\right)$ is the stochastic noise from the environment and/or model uncertainty. Equation (1) states that the level of target protein *i* is associated with its interacting proteins, basal level and stochastic noise. By augmenting the levels of protein *i* in *M* samples (M=23 in this study), *i.e.*, by letting $y_{i}={\left[\begin{array}{ccc} y_{i}\left(1\right) & \cdots & y_{i}\left(M\right) \end{array}\right]}^{⊤},\forall i=1,\cdots ,P$, Equation (1) can be written in vector form:

(2) $$y_{i}=\Phi_{i}\theta_{i}+\epsilon_{i}$$

where $\Phi_{i}=\left[\begin{array}{cccc} y_{1} & \cdots & y_{P} & 1 \end{array}\right]$, $\theta_{i}={\left[\begin{array}{cccc} \alpha_{i1} & \cdots & \alpha_{iP} & \beta_{i} \end{array}\right]}^{⊤}$, and $\epsilon_{i}={\left[\begin{array}{ccc} \epsilon_{i}\left(1\right) & \cdots & \epsilon_{i}\left(M\right) \end{array}\right]}^{⊤}$. The next step is to estimate the unknown $\theta_{i}$ in Equation (2) based on the microarray data. This can be achieved by solving a least squares optimization with linear constraints, as follows:

(3) $$\min_{\theta_{i}}{∥\Phi_{i}\theta_{i}-y_{i}∥}_{2}^{2}\;\text{such}\;\text{that}\;\beta_{i}\geq 0$$

The active-set algorithm [53] is used for parameter estimation.

#### 3.2.3. Model Order Detection and Identification

Since the PPIs in the candidate network were based on a wide variety of biological experimental conditions and computational predictions in databases, there was a large number of false positive PPIs. These had to be screened further using microarray data for CE strokes to obtain realistic networks for specific biological stroke stages. The Akaike information criterion (AIC) was used to select the true interaction model order (*i.e*., the real number of proteins interacting with protein *i*) [54]. For a protein interaction model for target protein *i* with order *L*, where L∈{0,⋯,P}, *i.e.*, *L* proteins interact with target protein *i*, the AIC value is calculated as follows:

(4) $$AIC_{i}\left(L\right)=\log\frac{∥\Phi_{i}{\hat{\theta }}_{i}-y_{i}{∥}_{2}^{2}}{M}+\frac{2L}{M}$$

where ${\hat{\theta }}_{i}$ is the solution of Equation (3). According to the theory of system identification [54], the true system order should minimize AIC value in Equation (4). By forward selection and backward elimination, the model order *L* with the lowest AIC value for the protein interaction model of target protein *i* can be obtained. After completing model order detection and identification, the estimated parameters were further tested for their significance using Student’s *t*-test with the null hypothesis is $\alpha_{ij}=0$ and a *p*-value threshold of 0.05. The following is pseudo-code we used for the model order test based on minimum AIC value. Details of the network construction can be found in the network construction section of supplementary files.
**Require:** candidate network, gene expression profiles at a specific stage **for all** protein *i* in the candidate network **do**  $y_{i}\leftarrow$expression profiles of protein *i*  $\Phi_{i}\leftarrow$expression profiles of all proteins interacting with protein *i* in the candidate network  **function** AICstepwise($\Phi_{i},y_{i}$)   Start with forward selection and after each candidate (other than the first one) is added to the model, perform backward elimination to see if any of the selected candidates can be eliminated without increasing the AIC value.   **return**
$\theta_{i}$  **end**
**function**  **function** Ttest($\Phi_{i},y_{i},\theta_{i}$)   Calculate *p*-value for each interaction activity $\alpha_{ij}$ in $\theta_{i}$ and delete if ≥0.05.   **return**
$\theta_{i}$  **end**
**function** **end**
**for**

Finally, by assembling the estimated parameters $\alpha_{ij},i,j=1,\cdots ,P$ into a matrix, the resulting PPINs at four stages can be represented as $N_{C}$, $N_{I}$, $N_{II}$ and $N_{III}$.

### 3.3. Network Analysis

#### 3.3.1. Functional Networks

To improve the capturing of essential information from the constructed networks, two different levels of analyses were used to explore the functional and molecular relationships at different stages of early stroke. First, the proteins in the constructed networks can be divided into several groups according to the PANTHER function classification system [55], based on their belonging functions. The functional networks at each stage consist of these enriched functions and the interactions between them. The interaction activity between two enriched functions is obtained by summing the interaction activities between member proteins of the two functions. The up- and down-regulated interactions between enriched functions can be observed by differentiating the functional networks of two stages.

#### 3.3.2. Core Networks

Second, due to the large size of the constructed networks, their essential components can be more effectively illustrated by a core network. A core network is defined as a network of core proteins whose interactions are similar to the principal eigen-interactions of the constructed networks. Singular value decomposition (SVD) is applied to determine the eigen-interactions $v_{i}$ by extracting the main features of the constructed networks. Given that *N* is the matrix representation of the network at different stages of CE stroke (*i.e*., $N=N_{C}$, $N_{I}$, $N_{II}$, or $N_{III}$ in this study), the SVD of *N* is:

(5) $$N=U\Sigma V^{⊤}$$

where *U* and *V* are unitary matrices and Σ is a diagonal matrix with singular values $\sigma_{i}$ of *N* on its diagonal. The eigen-interactions $v_{i}$ are the columns of *V*, *i.e.*, $V=\left[\begin{array}{ccc} v_{1} & \cdots & v_{P} \end{array}\right]$ with corresponding singular values $\sigma_{i}$ such that $\sigma_{1}\geq \sigma_{2}\geq \cdots \geq \sigma_{P}$. The percentage of the network explained by the *i*-th eigen-interaction can be calculated as follows:

(6) $$r_{i}=\frac{\sigma_{i}^{2}}{\sum_{i=1}^{P}\sigma_{i}^{2}}\times 100\%$$

We can then choose *M* principal eigen-interactions to meet a heuristic condition that will be application-dependent. In this study, we chose the smallest *M* such that $\sum_{i=1}^{M}r_{i}\geq 85\%$, which is conventionally used in principal component analysis. A core network can then be constructed by selecting proteins based on the similarity of their interactions to the principal eigen-interactions $v_{1},\cdots ,v_{M}$. The inner product between protein interactions ($\left[\begin{array}{ccc} \alpha_{i1} & \cdots & \alpha_{iP} \end{array}\right]$) and eigen-interactions ($v_{i}$) is used to evaluate the similarity of the interactions. Proteins with similarity above some threshold (>6 in this study based on the number of nodes in the resulting core networks) are called core proteins, and the network formed by the core proteins and the interactions between them is called the core network.

## 4. Conclusions

In this study, protein-protein interaction networks for four stages of stroke pathogenesis were constructed in a systems biology framework, based on multi-database mining, microarray data and protein interaction models. Functional classification and principal network projection were used to derive functional and core networks. Comparative network analysis was then used to investigate the underlying mechanisms of stroke pathogenesis at functional and protein levels. The configuration of enriched functions after stroke onset suggests a reasonable mechanism (Figure 4). Potential targets of miRNA and methylation regulations are proposed as potential therapeutic drug targets. Our results provide a direction for future study in stroke pathogenesis and treatment.

## Figures and Tables

**Table 1:** Number of proteins and interactions in four stage-specific constructed protein-protein interaction networks (PPINs) of CE stroke.

Stage	Number of Proteins	Number of Interactions
Control (C)	11,554	91,729
≤3 h (I)	9433	52,295
5 h (II)	9432	52,774
24 h (III)	9339	51,989

**Table 2:** Potential miRNA and methylation regulations in early CE stroke pathophysiology.

	Target Protein	Regulation Type	Function of Target Protein	Literature Validation
Lowered level	BCAT1	miR-21, 25, 140, 146a	cell growth	[31,32] and references therein
	AKAP12	miR-29b-1, 181a, 183, 335	cell growth	[31,32] and references therein
	DUSP14	miR-16, 26b	signaling pathway	[31,32] and references therein
	FECH	miR-16, 25, 124	heme synthesis	[31,32] and references therein
	H1F0	miR-181a, 494	histones	[31,32] and references therein
	TAGLN	miR-26b, 149	undetermined	[31,32] and references therein
	UBE2O	miR-328, 335	protein synthesis & turnover	[31,32] and references therein
	RPS4Y1*	miR-19b	protein synthesis & turnover	[31,32] and references therein
	SPP1*	miR-146a, 335	coagulation	[31,32] and references therein
	C4BPA	hypermethylation	complement system	[35]
	CD3G	hypermethylation	complement system	[36]
	DEPDC7	hypermethylation	protein synthesis & turnover	[38]
	FECH	hypermethylation	protein synthesis & turnover	[39]
	HLA-DQA1	hypermethylation	leukocyte activation	[40]
	NKX3-1	hypermethylation	protein synthesis & turnover	[42]
Elevated level	RHEB	miR-18a, 155	cell growth	[31,32] and references therein
	RPL27	miR-186	protein synthesis & turnover	[31,32] and references therein
	ACTA2	hypomethylation	inflammation	[34]
	CENPK	hypomethylation	cell growth	[37]
	HLA-DRB4	hypomethylation	Leukocyte activation	[41]

* Indicates the selected potential drug targets.

## Supplementary Materials

Supplementary materials can be found at http://www.mdpi.com/1422-0067/17/3/305/s1.
