# Supplementary material for: Key Immune Events of the Pathomechanisms of Early Cardioembolic Stroke: Multi-Database Mining and Systems Biology Approach
Source: Int J Mol Sci. 2016 Feb 27;17(3):305. doi: 10.3390/ijms17030305 (PMC4813168; doi:10.3390/ijms17030305)
Supplement: Supplementary file 1 [file ijms-17-00305-s001.zip › ijms-109897-Supplementary Materials/ijms-109897-supplementary.pdf]

Supplementary Materials: Key Immune Events of the Pathomechanisms of Early Cardioembolic Stroke: Multi-Database Mining and Systems Biology Approach

Chia-Chou Wu and Bor-Sen Chen

In this supplementary document, we described the exact procedures we used to construct networks and following analysis. Meanwhile, the corresponding code and data files were indicated in the context.

1. Microarray Data Retrieve and Preprocessing

The raw files (*.CEL) of gene expression following cardioembolic stroke are downloaded from Gene Expression Omnibus (GEO) database with the link: http://www.ncbi.nlm.nih.gov/geo/query/acc.cgi?acc=gse58294. To further process the raw files, library files (see Figure S1) are needed and downloaded from the Affymetrix®. GC Robust Multi-array Average-Empirical-Bayes (GCRMA-EB) background adjustment, quantile normalization, and median-polish summarization were then performed on the raw data (CEL files, see Figure S2) using MATLAB® (see the preprocessing.m in the supplementary zip file). By replacing the probe set ID with the gene symbol, we obtained 23,520 unique genes and their expression profiles at four time points (control, ≤3, 5, 24 h post-stroke) and 23 samples for each time points (see strokeUniGenes.mat in the supplementary zip file).

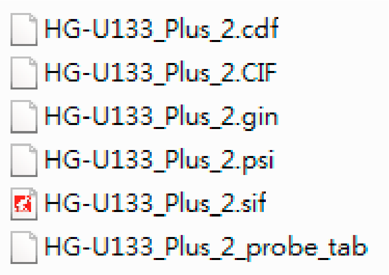

Figure S1. Library files downloaded from Affymetrix®.

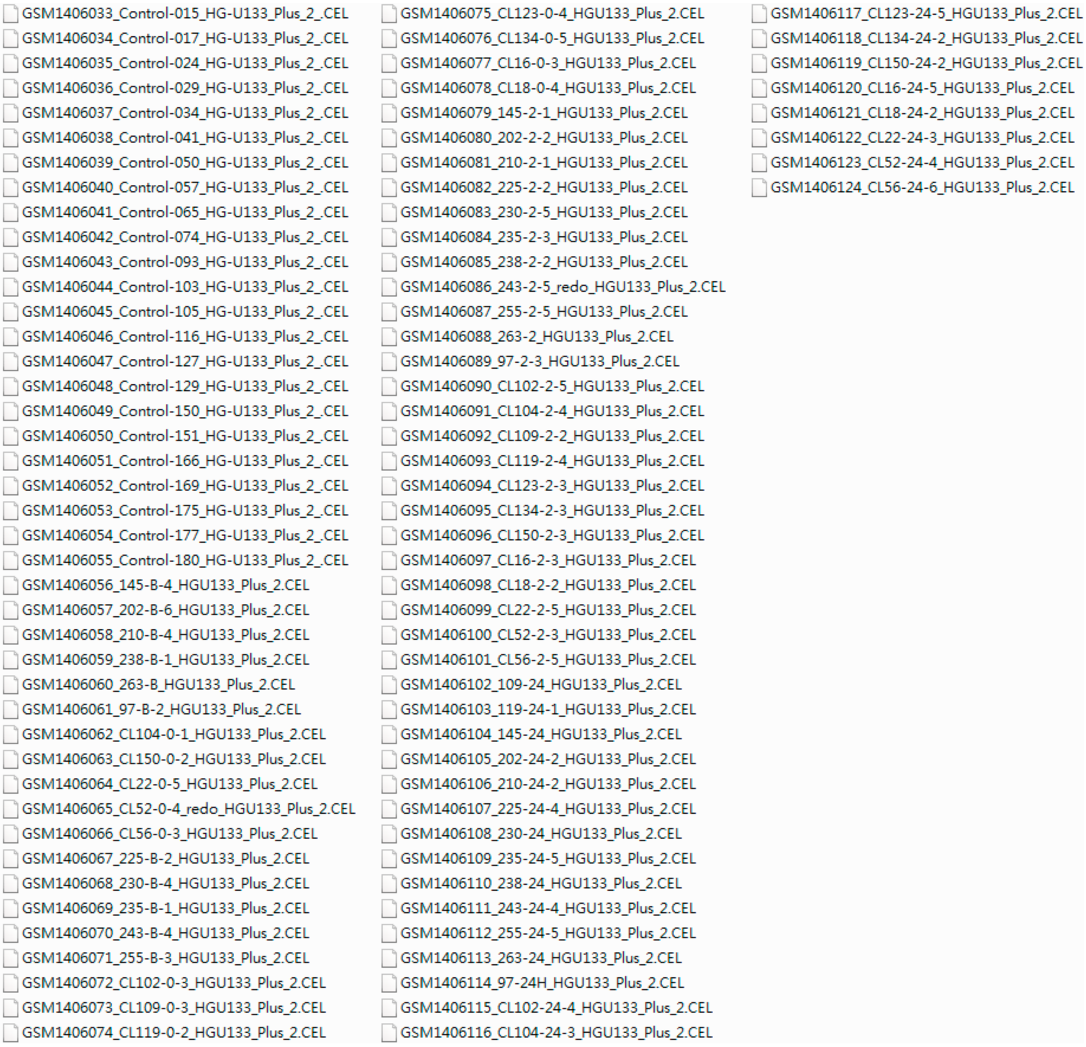

Figure S2. Raw files downloaded from GEO database.

2. Multi-Database Mining.

2.1. Data Retrieve

Ten protein-protein interaction (PPI) databases were used to construct the candidate PPI network (PPIN). The PPI information downloaded from the databases were further processed into two-column tables. For example, the PPI information downloaded from BioGRID is a 24-column table and each column is with its own meaning (see the BioGRID website or the link http://wiki.thebiogrid.org/doku.php/biogrid_tab_version_2.0 for details). We only extracted the 8th and 9th columns to form a two-column table. By the similar procedure, we downloaded PPI information from the ten databases mentioned in the Material and Methods section and obtained ten two-column tables. The ten two-column tables were integrated into one two-column table (see human_DBPPI.mat in the supplementary zip file) by removing replicated PPIs. There were 393,906 PPIs and 18,883 unique proteins in the resulting two-column table.

2.2. Candidate Network

Obviously, not all genes with expression profiles can be found in the PPI information from the databases and not all symbols in the two-column table had their expression levels were measured in the microarray. Therefore, we only considered the genes having expression profiles and PPI information. That is, we intersected 23,520 unique genes in the microarray data and 18,883 unique proteins in the two-column table to obtain the candidate network. There were 15,017 unique proteins

and 319,362 PPIs in the candidate network. Thus, for each protein in the candidate network, it had expression profiles from microarray and PPI information from databases.

3. Network Construction

3.1. Description

The candidate network constructed in the previous step was the initial network for the four stages corresponding to the four time points. By following the algorithm in the Material and Methods section, one PPIN was obtained for each stages. For each stage, we started from the same candidate network and the corresponding 23 microarray samples. Then, for each protein in the candidate network, its potential interactors and their expression profiles were used to find the minimum AIC value (see the following example for details). The netConstruction.m in the supplementary zip file is the main program to construct networks and the remaining m files (PPIconstruction.m, listToMatrix.m, matrixToList.m, AICfun_ForStepRegression_constrained.m, AICfun_backward_constrained.m, and pvalue_selection_constrained_gene.m) are the functions called by the main program. After running the main program, four PPINs were obtained and further analyzed.

3.2. Example

Here, we took a protein (A1BG) in the candidate network and its expression profiles in the control sample as an example to demonstrate the procedures of network construction (see Table S1 for summary of the procedures). The A1BG protein had nine potential interactors found in the candidate network. This means there are at most nine interaction activities and one basal level to be estimated. First, we assumed there are no interactions between A1BG and its nine interactors. In this situation, only one parameter, basal level, is estimated and the AIC value of this model is also calculated. Then, with forward selection, one of nine parameters is added back to the model and this will generated nine models and the corresponding AIC values. When the 6th interactor is added back to the model would give the minimum AIC value among the nine models. No back eliminations are performed after 1st forward selection. In the 2nd forward selection, we added one more interactor back to the model to see whether the AIC value can be decreased. We found when the 3rd interactor is added back to the model would further decrease the AIC value to −3.2230. Then, 1st backward elimination is performed to see whether the AIC value can be decreased by removing one interactor. We found no interactors are needed to be eliminated from the model to decrease AIC value. In 3rd forward selection, we found no interactors are needed to be selected into the model to decrease AIC. In the end, for A1BG protein, we found two interactors (3rd and 6th interactors) can give a model with the minimum AIC value. By iterating the procedure for each protein in the candidate network and the microarray data at each stage, the interactions of each protein at each stage which can minimize the AIC values can then be identified. By assembling the estimated interaction activities into a matrix, the resulting PPINs at four stages can be obtained.

Table S1. The AIC stepwise procedure for determining the order of the A1BG protein interaction model.

Step	Model	AIC Value
Initial	$y_i(m) = \beta_i + \epsilon_i(m)$	0.4556
1st forward selection	$y_i(m) = \alpha_{ik} y_k(m) + \beta_i + \epsilon_i(m)$, $k = 1, \cdots, 9$	
	When $k = 6$, the AIC value can be minimized.	−3.2079
2nd forward selection	$y_i(m) = \alpha_{ik} y_k(m) + \alpha_{i6} y_6(m) + \beta_i + \epsilon_i(m)$, $k = 1, \cdots, 5, 7, 8, 9$	
	When $k = 3$, the AIC value can be minimized.	−3.2230
1st backward elimination	$y_i(m) = \alpha_{ik} y_k(m) + \beta_i + \epsilon_i(m)$, $k = 3, 6$	
	No k are needed to be eliminated to decrease AIC value.	
3rd forward selection	$y_i(m) = \alpha_{ik} y_k(m) + \alpha_{i3} y_3(m) + \alpha_{i6} y_6(m) + \beta_i + \epsilon_i(m)$	
	k=1, 2, 4, 5, 7, 8, 9	
	No k are needed to be selected to decrease AIC value.	

4. Functional Network

The Gene List Analysis in PANTHER classification system [55] were used to find the enriched functions in the four constructed networks The statistical method used to conduct the overrepresentation test is the binomial test. The member proteins of each enriched function used to construct the functional networks and differentiation between the networks of different stages generated the differential networks given in Figures 2A, 3A, and 4A were given in the supplementary excel files (Tables S2–S5).

5. Cytoscape File

The cytoscape software was used to present the networks in the Figures 2, 3, and 4 and the corresponding files (figure2A.cys, figure2B.cys, figure3A.cys, figure3B.cys, figure4A.cys, and figure4B.cys) were given in the supplementary zip file.

6. miRNA–Target Database

miRTarBase [31] and ref. [44] and [45] were used to construct miRNA regulation in Table 2. Refs. [44] and [45] provided the miRNAs which were up- and down-regulated in strokes and miRTarBase provided the targets of the dysregulated miRNAs in refs. [44] and [45]. Comparing the targets found in the miRTarBase with our results on the changes of basal levels of proteins, we inferred the potential miRNA regulations and their targets in the constructed networks.
